# Change in preschoolers’ PTSD symptom severity predicted by caregivers’ emotional flooding and aggression

**DOI:** 10.1017/S0954579426101709

**Published:** 2026-07-09

**Authors:** Helena Her, Amy D. Marshall

**Affiliations:** 1 University of Pittsburgh School of Medicine, USA; 2 Virginia Polytechnic Institute and State University, USA

**Keywords:** Abuse, fathers, parenting, posttraumatic stress disorder, preschool children

## Abstract

Young children who experience symptoms of posttraumatic stress disorder (PTSD) rely heavily on caregivers for emotional support. Yet, caregivers who are overwhelmed (or “flooded”) by children’s emotional expressions may not provide effective responses, particularly if they also engage in parent-to-child aggression (PCA). Over 12 months, partnered caregivers (*N* = 448, *M*age = 33.80, 52.5% female, 62.7% non-Hispanic White) of a child age 3–5 years who was eligible for Head Start completed surveys and interviews to assess parental emotional flooding, PCA, and children’s trauma exposure and PTSD symptoms. Most children (89%) were exposed to potentially traumatic events, including 74% during the study. Male caregivers’ emotional flooding predicted maintenance of children’s PTSD symptoms over time, regardless of the degree of PCA engaged in. Female caregivers’ emotional flooding predicted maintenance of children’s PTSD symptoms only among those who engaged in high levels of PCA. Caregivers’ experience of emotional flooding may impact children in ways that maintain their PTSD symptoms, including modeling of ineffective emotion regulatory processes or children’s internalization of emotions to avoid negative parenting behaviors. Even at low levels of PCA, men’s emotional flooding may be impactful, potentially via parental withdrawal. Interventions addressing caregivers’ distress when managing children’s PTSD-related emotions are indicated.

The prevalence of trauma exposure among young children in the United States is substantial, with approximately 26% of children experiencing a potentially traumatic event (PTE) before they turn four years of age (Briggs-Gowan et al., 2010). In socioeconomically disadvantaged communities, the rates of trauma exposure are even higher, with estimates as high as 72% (Roberts et al., 2013). Such disparities may stem from the increased exposure of children in low SES families to greater parental and financial stress, community violence, and an inadequate support structure (Giovanelli & Reynolds, 2021). It is estimated that the annual lifetime cost of one type of trauma, child maltreatment, is between 428 billion (based on *substantiated* cases) and 2 trillion (based on *investigated* cases) dollars (Peterson et al., 2018).

Childhood trauma exposure can have significant long-term effects on individuals’ physiology and physical health, and the highly plastic early childhood brain appears to be especially sensitive to environmental stressors (Gunnar & Quevedo, 2007; Teicher et al., 2003). Thus, early traumatic experiences can lead to significant neurobiological changes, including changes to the structure and functionality of the brain, the hypothalamic–pituitary–adrenal axis, and the autonomic nervous system (Agorastos et al., 2018). Short term dysregulation of these systems may result in observable behavioral changes, while long-term dysregulation may result in the development of stress system disorders and may mediate the association between early trauma and long-term risk of disease vulnerability in adulthood (Agorastos et al., 2018; Beilharz et al., 2020).

One of the most predominant clinical concerns in relation to trauma exposure is the development of posttraumatic stress disorder (PTSD; Danese et al., 2020). Among young children, PTSD rates have been found to range from 26% among community samples (Levendosky et al., 2002) to 69% among clinical samples (Scheeringa & Zeanah, 1995). A recent meta-analysis (Woolgar et al., 2022) reported that, using developmentally appropriate diagnostic criteria, approximately 22% of preschool-aged children exposed to PTE(s) meet diagnostic criteria for PTSD, exceeding meta-analytic prevalence rates for older children and adolescents (16%). Child PTSD is commonly associated with poor psychosocial adjustment such as increased irritability, lower scores on cognitive measures, and poor academic performance (Bücker et al., 2012; De Young et al., 2011; Larson et al., 2017). Child PTSD symptoms have also been linked to greater challenges in developing and maintaining interpersonal relationships, including increased rates of peer rejection and victimization, and difficulty trusting caregivers (De Young et al., 2011; Goemans et al., 2023). Together, these effects of early trauma and PTSD can cascade into further long-term developmental challenges (Masten & Cicchetti, 2010).

## Parenting and children’s PTSD symptoms

Young children are highly dependent on their caregivers to provide them with a safe home environment and scaffold critical regulation skills (Eisenberg et al., 2010). Parental scaffolding of emotion regulation skills is particularly salient during the preschool years as children have limited coping mechanisms during this period and rely on their parents to support and comfort them (Kopp, 1982). Generally, parents may offer a safe, nurturing environment to process events that may be overwhelming for children to manage themselves (Laible, 2004). Supportive parental responses to children’s negative emotions may help children understand their emotions and teach them techniques to handle their emotions in the future (Eisenberg et al., 2010, Morris et al., 2007), whereas non-supportive parental responses may teach children that their negative emotions are inappropriate, inducing further dysregulation (Eisenberg et al., 1998). Positive parental emotion socialization may be especially important for children with elevated PTSD symptoms as they must regulate high levels of distress and emotional arousal (Fainsilber Katz et al., 2016; Overbeek et al., 2022), and difficulties with self-regulation likely contribute to the maintenance of PTSD symptoms via increased avoidance and emotional numbing (Villalta et al., 2018).

Children’s negative emotionality can be challenging for parents to regularly de-escalate, subsequently changing parenting behaviors (Oliver, 2015; Shewark et al., 2021) via a variety of possible mechanisms. First, parents may become overly cautious or protective of their children following trauma exposure, reflecting attempts to prevent their children from being traumatized again. Indeed, child trauma exposure has been linked to increases in parental monitoring (Bokszczanin, 2008, Henry et al., 2004). Second, having to frequently attend to their children’s emotional outbursts may cause parenting stress, caregiving burden, and diminished psychological resources needed for optimal parenting (Bussing et al., 2003; Vaughan et al., 2012). Finally, parents may experience secondary traumatization following their children’s trauma, causing parents’ own distress and avoidance of emotions as a trauma cues (Foa & Kozak, 1986). High levels of parental psychological distress, as well as depression and PTSD symptoms, following their children’s trauma exposure have been repeatedly observed (Holt et al., 2014; Wilcoxon et al., 2021). Importantly, Ward and colleagues (2024) recently found that maladaptive emotion regulation strategies among parents following their children’s trauma exposure were associated with greater child PTSD symptoms.

Parenting behaviors, both negative and positive, are salient to children’s adjustment, and may predict the persistence of children’s PTSD symptoms or act to buffer the link between trauma exposure and development of PTSD symptoms (Scheering & Zeanah, 2001). However, this research area is relatively small, lacks participation of male caregivers, and is almost exclusively cross-sectional, making it difficult to establish causal relations. Results across studies are also often conflicting. A recent meta-analysis reported substantial inconsistency across studies of parenting (e.g., parental warmth, hostility) and child PTSD symptoms, effectively resulting in small associations between child PTSD symptoms and both “positive” and “negative” parenting behaviors (Williamson et al., 2017). The use of broad constructs such as “positive” or “negative” parenting may provide some explanation for the mixed findings within the literature. Instead, it may be more useful to analyze children’s PTSD symptoms in relation to specific parenting behaviors that might be especially impactful as well as specifically targeted in clinical interventions.

## Parents’ emotional flooding

The construct of emotional flooding was first introduced by Gottman (1993) in the study of interpersonal, particularly couple, relationships. Gottman (1993) described it as the experience of another person’s negative emotional expressions as unexpected, intense, overwhelming, and cognitively disorganizing. Emotional flooding is not a specific emotional experience, but rather the experience of someone else’s emotional expressions as being overwhelming. Emotional flooding is theorized to disrupt cognitive and regulatory processing, affecting one’s ability to attend to a situation in a calm, organized manner. Frequent feelings of emotional flooding may condition a fight-or-flight response in which individuals seek to immediately terminate the overwhelming experience upon observing another person’s emotional expression. Such conditioning is thought to lead to emotional disengagement and relational isolation, with tendencies toward emotional flooding quickly developing into stable, trait-like methods for handling particular dyadic interactions (Gottman, 1993).

When applied to the parent–child domain, parents who tend to experience emotional flooding relative to their children’s emotional expressions may develop parenting styles and habits that interfere with their ability to effectively attend to their children’s distress and psychological needs (Slep & Heyman, 1998), including those that coincide with children’s experience of PTSD symptoms. For example, parents who find their children’s negative emotions to be overwhelming may not possess the ability to engage in effective scaffolding of their children’s self-regulatory skills. Indeed, parents who report relatively higher levels of parental emotional flooding have been found to engage in more permissive or inconsistent discipline (Del Vecchio et al., 2016; Lorber et al., 2016), potentially reflecting Gottman’s (1993) proposition that emotional flooding promotes disengagement from relational interactions. A flooding-induced absence of active, positive scaffolding of children’s regulatory capacities when they experience distress may thereby prevent recovery of children’s PTSD symptoms over time. Indeed, parents’ early negative trauma appraisals and encouragement of avoidant coping predict higher child PTSD symptoms six months post-trauma (Hiller et al., 2018).

At the same time, an important difference exists between the experience of emotional flooding in couple contexts and the experience of emotional flooding in parenting contexts. That is, whereas individuals can often escape their partners’ or other adults’ distress by disengaging and physically removing themselves from the situation, parents of young children cannot always employ the same techniques. Indeed, parents’ tendencies to experience emotional flooding with their children has been associated with engagement in more negative parenting behaviors such as harsh or aggressive parenting, which are thought to have developed as a means of quickly reducing the aversive experience (Del Vecchio et al., 2016; Lorber et al., 2016; Mence et al., 2014; Slep and O’Leary, 2007). However, because children’s expressions of distress are in some ways designed to elicit parental responsiveness to children’s needs (Gennis et al., 2022), children who come to expect parental flooding and associated ineffective or unhelpful parental behaviors may experience, and potentially express, even greater negative affect. Thus, the impact of parental emotional flooding on children’s PTSD symptoms may be amplified in negative parenting environments. Not surprisingly, harsh and/or aggressive parenting practices are associated with the severity of child PTSD symptoms, both during childhood (Valentino et al., 2010) and later in adulthood (Taillieu et al., 2016).

It is plausible that over time, parents’ emotional flooding may impair their ability to appropriately and reliably respond to children’s emotional needs and, thus, contribute to an environment that is more susceptible to escalation, unpredictability, or reduced emotional support (Carreras et al., 2019). In turn, children who have experienced such negative environments may be sensitized to their parents’ experience of emotional flooding and learn to routinely internalize, avoid, or mask their emotional experiences (Jones et al., 1998), thus contributing to the maintenance of PTSD (Foa & Kozak, 1986). Parent-to-child aggression (PCA), especially when broadly conceptualized, can serve as an especially impactful and behaviorally specific marker of negative parental environments. However, thus far, many of these topics have been examined separately, and there is a need to integrate them to study the potential effect of parents’ emotional flooding on children’s recovery from trauma. In particular, given the uniqueness of the parent–child relationship relative to couple relationships as well as the high prevalence of PCA within families of young children (Slep & O’Leary, 2007), it is especially important to examine whether the expected negative association between parental emotional flooding and children’s recovery from PTSD is amplified among families in which the parent engages in a relatively high level of PCA. To our knowledge, no existing studies have examined the potential association between parents’ emotional flooding and children’s recovery from PTSD, nor the potential moderating role of aggressive parenting behaviors. Doing so may spur additional real-time research designed to elucidate specific mechanisms of action (e.g., children’s expectation and fear of parental aggression, delayed learning of self-regulatory skills, increased negative affect in the context of parental unsupportive responses, increased internalization or avoidance of emotions).

## Research including mothers and fathers

Despite increasing involvement in child rearing by fathers in recent years (Bureau of Labor Statistics, 2024) and evidence linking increased paternal involvement with positive child outcomes (Yogman et al., 2016), fathers continue to be underrepresented in child psychopathology research (Schulz et al., 2023). Moreover, contemporary theories of gender similarities and differences in parenting, as well as associated empirical examinations, are largely lacking. Building on attachment theory (Bowlby, 1969), Paquette (2004) proposed that, in general, mothers and fathers often serve distinct roles for their children. Specifically, Paquette (2004) theorized that mothers commonly play a greater role in providing emotional support to children, whereas fathers play a greater role in providing authority, control, and encouragement of children’s exploration of unfamiliar environments (Van Lissa et al., 2019). Paquette (2004) argued that this simultaneous encouragement of risk and provision of structure and security by fathers promotes children’s emotion regulation in the context of novel challenges. In the context of child PTSD, it may be that mothers are especially able to support emotional processing of trauma stimuli and fathers are especially able to prevent behavioral avoidance of feared trauma-based stimuli.

These ideas align with a comprehensive review of parenting studies across 15 countries that concluded that, overall, mothers are more accepting, emotionally responsive, and supportive compared to fathers, who were more restrictive and harsher (Yaffe, 2023). Mothers also appear to be more perceptive of children’s emotional and behavioral changes (Trumello et al., 2023). Additionally, children appear to respond with reduced social inhibition and anxiety to fathers’ encouragement of risk taking, whereas the same encouragement by mothers increased children’s inhibition, potentially because such maternal behaviors conflicted with children’s expectations of maternal support and emotional safety (Majdandžić et al., 2014). These differences may explain Del Vecchio and colleagues’ (2016) finding that mothers experienced significantly higher levels of emotional flooding in parenting contexts than did fathers. At the same time, mothers tend to report higher levels of parenting stress than fathers (Trumello et al., 2023). This may reflect gender-based differences in sensitivity to children’s emotional experiences and concurrent demands to engage in a responsive and supportive manner along with generally greater parental responsibilities than fathers (Bureau of Labor Statistics, 2024), potentially fueling mothers’ greater tendency toward parental emotional flooding (Del Vecchio et al., 2016). When mothers’ parenting stress and overwhelm peak such that they cannot balance all such demands, they may begin to engage in aggression toward their children. Indeed, parenting stress when children are a year old is a strong predictor of mothers’ engagement in PCA at when children are three years of age (Le et al., 2017).

In the context of children’s PTSD, mothers’ provision of comfort during times of distress may benefit children’s self-regulation skills by scaffolding their emotional coping abilities, and mothers’ responsiveness may indirectly foster secure exploration and self-assertion by being attuned to their children’s needs and demands (Baumrind, 1991, Van Lissa et al., 2019). Likewise, fathers’ control may benefit children by providing clear structure for exploration and appropriate behavior, while supporting the down-regulation of negative affect consistent with social and/or family norms (Van Lissa et al., 2019). Altogether, mothers and fathers may form different relationships with their children, scaffolding different regulation skills that are key to recovery from PTSD and become especially important for preschool-aged children as they make the social transition into school settings (Morris et al., 2007). Yet, when parents become overwhelmed and flooded by their children’s affective expressions, they may be unable to provide the supports necessary to ensure emotional processing, minimization of avoidant coping, and healthy development of the emotion regulation skills necessary for recovery.

## The current study

The current study is designed to examine whether female and male caregivers’ experience of emotional flooding is associated with changes in preschool-aged children’s PTSD symptoms over time. We also examine whether the expected association between caregivers’ emotional flooding and children’s impaired recovery from PTSD is amplified within families in which the caregiver has engaged in relatively high levels of PCA. We hypothesized that (1) parents’ report of higher levels of baseline emotional flooding would be associated with the maintenance of (i.e., less decrease in) their children’s PTSD symptoms over time, and (2) parents’ aggression toward their children would moderate this relationship such that parents’ emotional flooding would predict less decrease in children’s PTSD symptoms to a greater degree among those who engage in higher levels of PCA. Given the dearth of research on fathering in general, as well as maternal versus paternal experiences of parental emotional flooding specifically, we tested models separately among female and male caregivers to explore whether similar or different patterns of results might emerge.

## Methods

### Participants

Participants (*N* = 448) included partnered caregivers of a child 3–5 years of age who was enrolled in, or eligible for, Head Start services. Participants were aged 20 to 64 years (*M* = 33.80, *SD* = 7.36), among whom 52.7% reported their sex as female and 47.3% reported their sex as male. In terms of ethnic and racial identity, most participants identified as non-Hispanic White (62.7%), followed by non-Hispanic Black (21.0%), more than one race (9.6%), Hispanic White (3.1%), and Hispanic Black (1.3%). An additional 2.1% of participants identified as Hispanic or non-Hispanic American Indian or Alaska Native, Asian, Native Hawaiian or other Pacific Islander, or Middle Eastern or North African. Most participants identified as heterosexual (81.9%), while 7.6% identified as bisexual, gay, or lesbian, 4.1% did not identify with any sexual identity, and 0.9% identified as asexual. No sexual orientation was reported by 3.3% of participants. Nearly all participants (98%) were in a heterosexual relationship. Roughly half (55.1%) of participants were not married to their partners. Most participants (88% of female caregivers and 82% of male caregivers) reported that the 3–5 year old child was their biological child. Participants resided in suburban (39.5%), urban (37.7%), and rural (21.2%) areas.

### Procedure

Data were obtained from the Children, Intimate Relationships, Conflictual Life Events, and Stress (CIRCLES) Study, a longitudinal project examining stress and aggression among caregivers of preschool age children. All procedures were approved by the Pennsylvania State University Institutional Review Board (STUDY00011179). Participants were recruited through community referral (e.g., Head Start programs), participant referral (i.e., snowball sampling), and social media advertisements throughout the Greater Pittsburgh Metro Region. To be eligible for the study, participants had to be (1) the primary caregiver or cohabitating partner of a primary caregiver of a child between the ages of three and five years who was enrolled in, or eligible for, a Head Start program, (2) at least 18 years old, and (3) able to speak English. Both partners within a couple must have been willing to participate. To accommodate less traditional relationships, cohabitation between partners was defined as spending three or more nights per week together. Head Start programs vary somewhat in eligibility criteria, but generally target children who reside in poverty (with some programs allowing a household income up to 130% of the poverty line), are in foster care, are homeless, or physically disabled. Over the course of approximately one year (*M* = 12.39 months, *SD* = 7.55 months), participants completed online baseline surveys, six semi-structured telephone interviews focused on family aggression, and an online follow-up survey. Variability across participants in time to study completion occurred, primarily due to delays in the completion of each portion of the study among a highly mobile sample of parents juggling multiple demands. Participants were compensated monetarily at each study segment, including a bonus for completing all study components, for a maximum of $400 per participant.

### Measures

#### Parents’ emotional flooding

Parents’ emotional flooding was measured using the Parental Flooding Scale (PFS; Slep & Heyman, 1998) at the beginning of the study. The PFS is a 15-item scale that has shown satisfactory factorial validity, concurrent validity, internal consistency, and test–retest stability (Del Vecchio et al., 2016). Example items include, “I find my child’s distress to be overwhelming,” “My child tends to explode without any warning signs,” and “I can’t think straight when my child is upset with me.” Caregivers rate items using a five-point scale, with 1 indicating “almost always” and 5 indicating “never.” Responses were reverse-coded and summed into a total score such that higher scores reflect greater emotional flooding. In the current study, the scale was internally consistent among women (*α* = 0.944) and men (*α* = 0.935).

#### Child trauma exposure

Child trauma exposure was measured using the Traumatic Events Screening Inventory- Parent Report Revised (TESI-PRR, Ghosh-Ippen et al., 2002) at the beginning of the study and an abbreviated version was administered at follow-up to assess children’s exposure to PTEs during the course of the study. The TESI-PRR is a revised version of the Traumatic Events Screening Inventory for Children (TESI-C, Ribbe et al., 1996) that assesses children’s (aged 0 to 6) lifetime trauma exposure from a caregiver’s perspective. The TESI-PRR has shown satisfactory reliability (Ford et al., 2000) and convergent validity (Berent et al., 2008). The TESI-PRR consisted of 24 items. An example item is: “Has your child been in a serious accident where someone could have been (or actually was) severely injured or died?” Each item was answered with “yes,” “no,” and “unsure” options.

#### Child PTSD symptoms

Child PTSD symptoms were measured using the Child Stress Disorders Checklist-Short Form (CSDC-SF; Enlow et al., 2010; Saxe et al., 2003) at the beginning of the study (baseline) and at follow-up. The CSDC-SF is a revised version of the 36-item Child Stress Disorders Checklist that has demonstrated internal consistency, inter-rater and test–retest reliability, and concurrent, discriminant, and predictive validity comparable to the full scale (Enlow et al., 2010). The CSDC-SF consisted of 4 items that caregivers rate relative to the child’s most stressful PTE (or group of related events if similar events occurred repeatedly). An example item is: “Child gets very upset if reminded of the event(s).” Additional items measure physical complaints when reminded of the event(s), avoidance of doing things that remind child of the event(s), and startling easily. Caregivers rated items using a three-point scale, with 0 indicating “not true” and 2 indicating “very true.” Responses were summed into a total score. At baseline and follow-up, internal consistency reliability of male caregivers’ reports of child PTSD symptoms were acceptable (*α* = 0.703 and 0.778, respectively). Internal consistency of female caregivers’ reports were also acceptable at follow-up (*α* = 0.784), but less so at baseline (*α* = 0.516). This lower level of internal consistency is not expected for this measure, but also is not unusual for a 4-item scale. Additionally, for female caregivers’ baseline reports, the mean inter-item correlation (*r* = .21) fell in the acceptable range for a brief measure (Briggs & Cheek, 1986) and, because we averaged male and female caregivers’ scores, error associated with female caregivers’ baseline reports should be minimized.

#### Parent-to-child aggression

PCA was measured using the Children, Intimate Relationships, and Conflictual Life Events (CIRCLE) Interview (Marshall et al., 2017). The CIRCLE Interview utilizes an event history calendar method to assess incidents of psychological and physical aggression between partners and toward children, though the current report focuses only on information regarding PCA. Estimates of PCA behavior frequencies from the CIRCLE Interview have demonstrated convergent, discriminant, structural, predictive, and incremental validity over more commonly used measures of PCA as well as a higher degree of inter-partner reporting concordance. Further, reports of PCA have been shown to not be influenced by repeated testing, social desirability, or attempts to avoid aggression because of study participation (Marshall et al., 2017).

Twelve interviewers (clinical psychology graduate students and research staff) were trained to administer the interview and respond to participant distress and disclosures using a behaviorally based manual and interview protocol, role plays, group discussions, and supervised live interviews. Participants were provided a list of standard aggressive behaviors and were asked to consider additional seemingly aggressive acts not listed. The context of participants’ descriptions was used to help interviewers determine whether behaviors constitute aggression, defined as non-playful behaviors that are threatening or forceful in nature. Participants were also provided with a calendar to note the days being assessed (the prior four weeks), days of personal significance (e.g., holidays, illnesses), and days of no face-to-face contact with their partners and children. Using these events to aid memory, participants worked backward in time to review all incidents of psychological and/or physical aggression that occurred during the prior four weeks. For each aggressive incident, participants reported the conflict topic, as well as the order, victim, and perpetrator of each aggressive behavior. They also identified “regular patterns” of aggression and, if present, reported the usual conflict topic, frequency of the pattern (with start and end dates if it did not occur continuously), and the order, victim, and perpetrator of each aggressive behavior. Administration of the full interview and debriefing took approximately 45 minutes.

A total PCA perpetration score was computed by summing the number of aggressive behaviors that the participant engaged in toward the age 3–5 years target child across all incidents. For “regular patterns,” the number of behaviors within a typical incident was multiplied by the frequency of the pattern during the interview period (i.e., the prior four weeks). A final PCA score, reflective of the total number of PCA behaviors the participant engaged in toward the target child during six non-consecutive months of the study, was obtained by summing participants’ PCA behaviors across all interviews.

### Statistical analyses

Prior to analyses, data were cleaned to ensure accurate measurement, avoid violations of analytic assumptions, and maximize use of both caregivers’ data. First, children were identified as having been exposed to a PTE at baseline and during the study period if either caregiver reported such exposure. For children who both caregivers reported no PTE exposure at baseline, they were assigned a baseline PTSD symptom severity score of zero. Children for whom both caregivers reported no PTE exposure at baseline or during the study period were excluded from the analyses. For each child, the mean of their caregivers’ reports of PTSD symptom severity was used in order to minimize reporter bias; if only one caregiver provided a report, that caregiver’s report was used. Representing the dimensional nature of PTSD symptoms (Broman-Fulks et al., 2006), continuous PTSD symptom severity scores were used. Within each gender, one statistical outlier on each measurement of child PTSD symptom severity and two statistical outliers on emotional flooding were truncated to the next highest score. Due to excessive skew, PTSD symptom severity scores at baseline and follow-up were log-transformed.

Complete data to address the study aims was available for 322 (72%) participants, including 170 women and 152 men. Missing data were primarily a function of 88 (20%) participants who did not provide data on child PTSD symptom severity at the follow-up assessment, mostly due to attrition prior to the end of the study. When examined separately by participant sex, participants who provided complete data did not differ significantly from those who did not provide complete data on any of the primary study variables (all *ps* > .231). Multiple imputation, including all primary study variables and covariates, was used to create 10 complete data sets reflective of uncertainty due to missing data. These data sets were each analyzed, and results were pooled across data sets. Given study hypotheses, and to avoid issues of dependency of partners within couples, all analyses were conducted separately by participant sex.

Study hypotheses were tested using stepwise regression analyses conducted separately by participant gender. In each analysis, log-transformed child PTSD symptom severity at the follow-up assessment served as the dependent variable. Log-transformed child PTSD symptom severity at the baseline assessment was entered at step one so that results could be interpreted as change in child PTSD symptom severity during the study period (i.e., from baseline to follow-up). Gender of the target child and number of children in the home were included as covariates to account for potential differential reports of PTSD symptom severity based on child gender and the possibility that caregivers with more children in the home may not report child psychological distress as accurately as caregivers with fewer children in the home (Seiffge-Krenke & Kollmar, 1998). Child PTE exposure during the study period as well as the time (i.e., number of weeks) that participants were in the study (from baseline to follow-up) were also entered as covariates at step one. At step two, caregivers’ report of their emotional flooding and engagement in PCA were entered. Step three included the interaction between caregivers’ emotional flooding and engagement in PCA. To examine robustness of results, regression analyses were re-run excluding statistically nonsignificant covariates.

## Results

Participants reported an average of 2.6 children residing in the home, with 53% of target children being girls. Among children for whom data were available from either caregiver, 89% (*n* = 162) were exposed to at least one PTE during their lifetimes, and 74% (*n* = 136) were exposed to at least one PTE during the course of the study (*M* = 12.39 months). Children who were exposed to a PTE during the study exhibited greater PTSD symptom severity at follow-up than children who were not exposed during the study, *t* (224) = −5.144, *p* < .001, *d* = 0.824). Female and male caregivers did not differ significantly in their reports of child PTSD symptom severity at baseline (*t* = 0.790, *p* = .431, *d* = 0.059) or follow-up (*t* = 0.873, *p* = .384, *d* = 0.077).

Among female caregivers with PCA data, 154 (84%) reported engaging in PCA during the six months assessed, with an average of 24.19 (*SD* = 30.80) acts engaged in. Among male caregivers with PCA data, 120 (73%) reported engaging in PCA during the six months assessed, with an average of 21.14 (*SD* = 31.75) acts engaged in.

Descriptive statistics and bivariate correlations among primary study variables (among caregivers of trauma exposed children) are presented in Table 1. Compared to female caregivers, male caregivers reported significantly less emotional flooding (*t* = 2.371, *p* = .018, *d* = 0.240). Female and male caregivers reported mostly similar levels of engagement in PCA (*t* = 1.556, *p* = .121, *d* = 0.167). Among female and male caregivers, child PTSD symptom severity at baseline and follow-up assessments were significantly intercorrelated, and each significantly correlated with parents’ emotional flooding. Neither female nor male caregivers’ engagement in PCA during the study period was significantly correlated with child PTSD symptom severity at baseline or follow-up. Additionally, female, but not male caregivers’ experience of emotional flooding was significantly correlated with their engagement in PCA.

**Table 1.** Descriptive statistics and bivariate correlations among primary study variables

	*M*	*SD*	1.	2.	3.	4.
*M*	–	–	0.32	0.31	29.01	15.47
*SD*	–	–	0.47	0.42	10.09	28.71
1. Child PTSD at follow-up	0.35	0.49	–	0.23**	0.25**	−0.13
2. Child PTSD at baseline	0.32	0.41	0.22**	–	0.20**	−0.00
3. Parental emotional flooding	31.55	11.01	0.19*	0.20**	–	−0.10
4. Parent-to-child aggression	20.36	29.60	0.10	−0.04	0.15*	–

*Note*. Female caregivers’ data are below the diagonal; male caregivers’ data are above the diagonal. Child PTSD symptom severity scores represent the couple mean and are log-transformed.

** *p* < .01, *p < .05.

**Figure 1.**
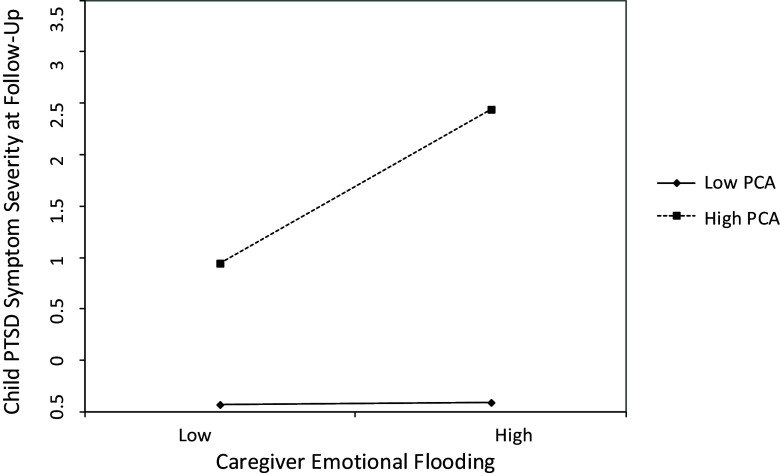
Female caregivers’ emotional flooding predicts their children’s PTSD symptom severity at 12-month follow-up (controlling for children’s baseline PTSD symptom severity) among caregivers who engaged in relatively higher levels of parent-to-child aggression (PCA) during the study, but not those who engaged in relatively low levels of PCA.

Results of stepwise regression models predicting child PTSD symptom severity at the follow-up assessment are presented in Table 2. Among female and male caregivers, child PTSD symptom severity at baseline as well as child exposure to a PTE during the study period significantly predicted child PTSD symptom severity at the follow-up assessment. After controlling for these variables as well as the number of children in the home, the target child’s gender, and the amount of time between baseline and follow-up assessments, among female caregivers, the interaction between their engagement in PCA and their experience of emotional flooding significantly predicted children’s PTSD symptom severity at follow-up. Simple slopes analyses indicated that emotional flooding predicted child PTSD symptom severity at +1 *SD* of PCA (*t* = 3.071, *p* = .002) but not at −1 *SD* of PCA (*t* = 0.333, *p* = .739). As displayed in Figure 1, children experienced more of an increase (or less of a decrease) in PTSD symptoms over the course of the study if their female caregivers experienced greater emotional flooding and engaged in relatively more PCA. Among children with female caregivers who engaged in relatively little PCA, the caregivers’ degree of experienced emotional flooding was unrelated to the children’s PTSD symptoms severity at follow-up. Among male caregivers, after controlling for child PTSD symptom severity at baseline, child exposure to a PTE during the study period, and each of the additional covariates, male caregivers’ experience of emotional flooding significantly and positively predicted child PTSD symptom severity at follow-up, whereas their engagement in PCA and the interaction between their engagement in PCA and their experience of emotional flooding did not significantly predict child PTSD symptom severity at follow-up. Because the covariates of number of children in the home, the target child’s gender, and the amount of time between baseline and follow-up assessments did not predict in the models for female or male caregivers, each model was re-run without these covariates to examine robustness of results. In both models, the pattern of effects remained the same.

**Table 2.** Prediction of child PTSD symptom severity at follow-up

	Female caregivers				Male caregivers			
	*b*	*SE*	*B*	*t*	*b*	*SE*	*B*	*t*
Constant	−0.050	0.154		−0.24	−0.186	0.164		−1.13
Child PTSD symptoms at baseline	0.224	0.088	0.197	2.56*	0.216	0.090	0.198	2.40*
Child PTE exposure during study	0.308	0.100	0.243	3.09**	0.226	0.100	0.184	2.26*
Number children in home	−0.039	0.027	−0.109	−1.45	−0.023	0.029	−0.061	−0.78
Target child gender	0.002	0.071	0.002	0.03	0.011	0.072	0.012	0.16
Time in study	0.002	0.001	0.114	1.42	0.002	0.001	0.114	1.29
Parent-to-child aggression (PCA)	0.001	0.001	0.044	0.53	−0.001	0.002	−0.042	−0.33
Parental emotional flooding	0.001	0.004	0.030	0.35	0.008	0.004	0.172	2.05*
PCA × parental emotional flooding	0.067	0.030	0.200	2.27*	0.001	0.055	0.002	0.02

*Notes*. PTE = potentially traumatic event; target child gender is coded 0 = girl, 1 = boy; time in study represents the number of weeks between completion of baseline and follow-up measures.

** *p* < .01, *p < .05.

## Discussion

Building upon prior research that established the importance of parents’ emotional flooding (Del Vecchio et al., 2016, Lorber et al., 2016; Mence et al., 2014; Slep and O’Leary, 2007), the current study was designed to examine the potential impact of male and female caregivers’ emotional flooding and aggressive behavior on their preschoolers’ PTSD symptom severity over time. This study is one of few to examine longitudinal associations between female *and* male caregivers’ parenting and change in their children’s PTSD symptoms, including examination of both what parents experience (i.e., emotional flooding) and what they do (i.e., engagement in aggression). This study also utilized a novel interview method to collect data about PCA, allowing us to capture precise estimates of the number of aggressive behaviors that occurred during a designated time period. Lastly, this study highlights the ubiquitous nature of childhood trauma within low-income, at-risk families, documenting uniquely high rates of lifetime and past-year exposure to PTEs (Briggs-Gowan et al., 2010; Roberts et al., 2013).

Among both female and male caregivers, the tendency to experience relatively more parental emotional flooding predicted the maintenance of their children’s PTSD symptoms over the course of the study (approximately one year on average). This was the case among male caregivers regardless of the degree of PCA that they engaged in, whereas female caregivers’ emotional flooding predicted maintenance of child PTSD symptoms only among those who engaged in relatively higher levels of PCA. These results suggest that higher PCA from female caregivers may amplify the impact of emotional flooding, serving to maintain children’s PTSD symptoms. It is possible that children of female caregivers who tend to engage in PCA learn to fear their caregivers’ emotional flooding because that caregiver cannot be trusted to regulate their emotions without negative consequences for the child. Without the presence of a secure parental relationship to experience and express their emotions in, these children may resort to internalization or avoidance of emotions following fear structure activation (Foa & Kozak, 1986). Children who regularly witness high levels of expressivity of negative emotions (e.g., anger, hostility, sadness) from their caregivers may also learn to internalize, avoid, or mask their emotional experiences in an attempt to regulate their experience of fear and to avoid negative parenting behaviors (Jones et al., 1998). Moreover, in addition to avoidance being a known mechanism of PTSD maintenance over time (Marx & Sloan, 2005; Shipherd & Beck, 2005), internalization of emotions in young children, in particular, has been linked to worse psychopathological symptoms (Compas et al., 2017). This interpretation aligns with previous research that linked parents’ emotional flooding to negative, harsh, and aggressive parenting behaviors (Del Vecchio et al., 2016; Lorber et al., 2016; Mence et al., 2014; Slep and O’Leary, 2007). However, because emotional flooding and PCA were measured at the between-person level and at separate time points, we cannot conclude that PCA and emotional flooding ever occurred simultaneously.

Interestingly, male caregivers’ experience of emotional flooding predicted the maintenance of their children’s PTSD symptoms regardless of the degree of PCA they engaged in. Because men tend to use fewer emotion regulation strategies compared women (Nolen-Hoeksema, 2012), men may be especially likely to experience difficulties regulating their emotions or behavior when flooded by their children’s emotional expressions, which can be frightening to children and/or serve as a poor model for the regulation of PTSD symptoms. That is, caregivers who experience higher levels of emotional flooding and are less able to effectively regulate their emotions may model to their children ineffective methods of regulation that serve to maintain PTSD symptoms. Indeed, children commonly reference and model their emotions through observing their parents’ emotional expressions and regulation strategies (Eisenberg et al., 1998; Schoppmann et al., 2019). Among male caregivers, withdrawal or non-aggressive expression of negative emotions may have been similarly impactful in terms of their children’s maintenance of PTSD symptoms. In fact, verbal and non-verbal negative expressions and emotional withdrawal from male caregivers have been linked to children’s emotional and behavioral dysregulation, though not specifically children’s PTSD symptoms (Chen et al., 2023; Foster et al., 2007; Gallegos et al., 2017). Additionally, the experience of emotional flooding may be especially likely to hinder male caregivers’ ability to effectively respond to their children’s needs (Nolen-Hoeksema, 2012) and, over extended periods of time, parental distress can maintain children’s stress reactions (Havighurst & Kehoe, 2017).

Like much of the field of parenting, more research is warranted to understand the observed gender differences in the current study. In addition to gender differences in the results of the primary analyses, on average, female caregivers reported higher levels of the experience of emotional flooding than did male caregivers, replicating findings of Del Vecchio and colleagues (2016). Despite fathers becoming increasingly involved in caretaking in the last few decades, on average mothers still report spending more time caring for and nurturing their children (Bureau of Labor Statistics, 2024), and mothers tend to be more sensitive and able to recognize children’s distress (Trumello et al., 2023; Yaffe, 2023). Fathers’ caregiving interactions tend to differ qualitatively from mothers’, skewing toward play rather than emotional caretaking (Craig, 2006), which may leave fathers with a less developed repertoire of non-aggressive interpersonal regulatory strategies. In this context, father’ engagement in PCA may reflect readily available means of terminating negative parent–child interactions. For mothers, engagement in PCA may be a marker of a degree of caregiver burden and parenting stress that has become unmanageable. In a less stressed environment, mothers may be able to overcome their experience of emotional flooding to still provide supportive parenting, but among highly stressed mothers such an ability may become untenable. To support this proposition, empirical examination of gender differences in caregivers’ ability to engage in supportive parenting that effectively scaffolds children’s emotion regulation while experiencing different levels of emotional flooding is needed.

Even in the case of PTSD, which appears to be largely a function of environmental exposure and context (Ozer et al., 2003), when studying parents and children, possible genetic influences must be considered. Importantly, the field of behavior genetics has taught us to also consider possible evocative effects of children’s characteristics and behavior on parents (Scarr & McCartney, 1983). For example, might greater child PTSD symptoms elicit greater emotional flooding and engagement in PCA among caregivers, as previously found in regard to harsh discipline among children with behavior problems (Choe et al., 2013; Pu & Rodriguez, 2021; Shewark et al., 2021). Although the current study does not allow for consideration of genetic influences, the longitudinal design provides some evidence that evocative effects of children on parents is not a likely explanation for the current results. Although it would have been ideal to measure parents’ emotional flooding at both baseline and follow-up in a cross-lagged panel design, prediction of change in child PTSD symptoms by caregivers’ baseline emotional flooding helps to establish a directional relationship. Additionally, female and male caregivers’ engagement in PCA was not associated with children’s PTSD symptoms at follow-up (or at baseline), suggesting that children’s PTSD symptoms did not lead caregivers to engage in PCA. However, additional longitudinal research is necessary to test these assumptions.

The results of this study should be considered in the context of additional research design limitations. First, these results cannot be generalized to all young children as this study was conducted with families recruited due to being enrolled in, or eligible for, a Head Start program in a select geographical region. Thus, replication with greater ethnic diversity and income variability is needed to understand the generalizability of the findings. Second, although we utilized both caregivers’ reports of child PTE exposure and PTSD symptoms as well as interviewer-validated reports of PCA, ultimately this study relied on self-report measures from caregivers. Moreover, although aggression interviews were subjected to routine supervisory oversight, they were not recorded, thus preventing examination of inter-rater reliability. Additionally, the internal consistency of female caregivers’ reports of child PTSD symptoms at baseline was less than ideal. We minimized the potential impact of measurement error by averaging male and female caregiver reports, but this issue provides additional reason to replicate the findings. Future studies should utilize multiple measures and multi-informant responses for all measures. Thus far, all studies, including our own, that measure parents’ emotional flooding have used self-report measures, but an at-home or laboratory-based caregiver–child assessment designed to invoke variation in child affect may offer further insight. Lastly, this study included only (broadly defined) coupled caregivers. It is possible the effects of PCA on the association between parental emotional flooding and children’s PTSD are even more pronounced in single caregiver homes, where there is less opportunity for buffering or positive emotion socialization from a more warm and regulated parent. Oppositely, it is also possible that when required to do most of the parenting, single caregivers of both genders become more similar, reducing the gender differences effects observed in our study (Dufur et al., 2010).

The results of this study suggest that addressing the feelings of distress and overwhelm caregivers may feel when their children express emotions is critical to helping children recover from PTSD. Interventions directed at helping parents experience less emotional flooding and to regulate their emotions, such as teaching cognitive reappraisal techniques, may be especially helpful (Kohlhoff et al., 2016). In addition to female caregivers, it is critical that such intervention efforts involve male caregivers, who have long been overlooked in research and interventions for children (Schulz et al., 2023). Children’s emotion regulation skills undergo rapid development during the preschool years (Denham, 1998; Denham & Kochanoff, 2002), and caregiver reactions to children’s negative emotional expressions may be especially impactful at this time, serving as a uniquely powerful window of opportunity to help children recover from PTSD. Additionally, the preschool years may be a sensitive period for the impact of PTEs (Teicher et al., 2003). Given this juncture of rapid development, increased risk, and sensitivity to environmental input, it is a critical timepoint for intervention efforts.

## Data Availability

The data, code, and associated materials for this study are currently available upon request from the corresponding author. The full data set and associated code and materials will be made publicly available at the completion of the parent project.

## Pre-registration statement

This research was not pre-registered as we did not expect to observe such high rates of child trauma exposure and PTSD symptom severity.
